# Lectures on Bright's Disease

**Published:** 1889-06

**Authors:** 


*Lectures on Bright's Disease.* By ROBERT SAUNDBY, M.D. Edin., F.R.C.P. Bristol: JOHN WRIGHT & Co. London: HAMILTON, ADAMS & Co. 1889.

It is a genuine pleasure to be able to say that a book is a good one. Of the vast quantities of medical literature published yearly a large proportion is devoid of solidity. Without hesitation we can say Dr. Saundby's book is both solid and good. Of the three sections into which the work is divided, the first on General Pathology contains chapters on Albuminuria, the Pathology of Dropsy, Cardio-vascular Changes, the Pathology of Uræmia, and some other subjects, all extremely well treated and the information brought up to date; in addition to which, a full and excellent bibliography of each special topic is added at the end of each chapter. In the suggestive chapter on Albuminuria we observe no allusion to Adams's observations on this point. Probably they are too recent to be incorporated into the work. The second section is composed of a detailed account of the clinical examination of the Urine in Health and Disease, in which full instructions are given to recognise by appropriate tests every substance of importance found in the urine whether normal or morbid. This, we think, is the best brief practical treatise we have seen on this subject, and will be of use to every practitioner and student. These two first sections of the book we cannot too highly commend. The third section, so far as it relates to the classification of the varieties of Bright's disease, we confess to finding less satisfactory. Dr. Saundby's etiological classification is into Febrile Nephritis, Lithæmic Nephritis, and Obstructive Nephritis. Setting aside the cases belonging to the last of Dr. Saundby's divisions, which mainly corresponds to the so-called "Surgical Kidney" consequent upon stricture, enlarged prostate, and other mechanical obstructions, the main bulk of cases of Bright's disease will come to be ranged under the other two heads, as lardaceous degeneration not complicated by nephritis Dr. Saundby does not consider a form of the malady. It will happen that in a very large number of instances the practitioner will be unable to discover either a past febrile process or present lithæmia to set down as the cause of the particular case he may be investigating; and must then, like Dr. Saundby on page 250, append a note of interrogation to the class to which the case must be assigned even when an autopsy has been performed on the patient. The etiological question has in the treatment of a case not so much to be considered as the pathological condition of the kidney; and in hospital practice especially it is often quite hopeless to attempt to discover the etiology, or to use it as a working hypothesis when supposed to have been found. Dr. Saundby has devoted an infinity of labour and study to the question; but in making this new arrangement of the modifications of Bright's disease we cannot think that he has dissipated any of the obscurity and difficulty which often surround the differential diagnosis in this disease, but, on the other hand, has rather rendered the subject more complicated than before. Dr. Saundby's admirable book is worth diligent perusal from cover to cover; and, except upon this one point of classification, there is nothing better extant in our language upon the subjects therein treated.

